# *Cancer Heterogeneity and Plasticity* — A new journal dedicated to understanding cancer cell states and interactions with the tumor microenvironment

**DOI:** 10.47248/chp2401010001

**Published:** 2024-07-18

**Authors:** Dean G. Tang

**Affiliations:** Department of Pharmacology & Therapeutics, Roswell Park Comprehensive Cancer Center, Buffalo, NY 14263, USA

Cancer claims a high mortality worldwide. The American Cancer Society estimates more than 2 million new cancer cases in the U.S. in 2024 [1]. Despite significant advances in oncology drug development and clinical approval in the past 25 years, especially in targeted therapeutics such as inhibitors of receptor tyrosine kinases (RTKs) and mutated oncogenes (e.g., K-Ras) and immuno-oncology (IO) drugs [2], >600,000 cancer-related deaths are projected for 2024 in the U.S. alone [1]. In addition, there are currently >18 million cancer survivors in the States, who would always have the worriment of cancer recurrence in the back of their mind. Why is cancer such a lethal and recalcitrant disease? As cancer is an extraordinarily complex ecosystem and represents an abnormally developed organ [3] and most cancer drugs target specific deregulated signaling drivers or pathways that operate in cancer cells, our failure in controlling and curing cancer is intimately related to cancer cell heterogeneity and plasticity, the scientific premise that has motivated us to launch the journal *Cancer Heterogeneity and Plasticity* (CHP).

Cancer cell heterogeneity, observed for many decades in both experimental and patient tumors, is manifested as variegated subpopulations of cancer cells that possess distinct phenotypic and functional properties, heterogeneously express the therapeutic target, and exhibit differential responses to anticancer drugs. For example, most cancers harbor the bulk ‘differentiated’ cancer cells (that express high levels of differentiation markers of the normal cells in the organ) as well as less mature cancer cells with many stem/progenitor cell properties called **cancer stem cells (CSCs)**. There is substantial evidence, in multiple cancers, that advanced, metastatic and therapy-resistant tumors have higher cancer stemness that is associated with increased aggressiveness and CSC abundance and/or state [4]. Most CSCs may lack or under-express the differentiation molecules targeted by the mainstay cancer drugs used in the clinic, e.g., androgen receptor (targeted by ADT/enzalutamide) in prostate cancer, estrogen receptor (targeted by tamoxifen and fulvestrant) in breast cancer, RTKs (targeted by tyrosine kinase inhibitors (TKIs)) in multiple epithelial cancers, and PD-L1 (targeted by immune checkpoint inhibitors or ICIs such as Atezolizumab, Avelumab and Durvalumab) in solid tumors. Cancer cell heterogeneity is also reflected, prominently, in a different aspect, *i.e.*, tumors harbor both proliferative and mitotic cells as well as **dormant** (**quiescent or slow-cycling**) cancer cells [5], which conceivably will be more resistant to general chemotherapeutic drugs and ionizing radiation that selectively kill cells in the cell cycle. Recent studies have also evinced a population of **polyploid giant cancer cells (PGCCs)**, which, interestingly, may also be dormant and share both phenotypic and functional features with CSCs [6-8]. Both treatment-naïve and treatment-failed tumors contain **senescent/senescing** and **EMT/EMT’ing** cancer cells that are undergoing or have completed the processes of cellular senescence and epithelial-mesenchymal transition (EMT), respectively. Studies in the past decade suggest that cancer cells transiting the mesenchymal and even the senescent [9,10] process also possess CSC traits and may preferentially survive drug treatment. As cancers, especially solid tumors, are metabolically heterogeneous, there also exist **glycolytic**, **hypoxic** and **acidic** cancer cell subpopulations. Once tumor cells break away from the primary site, enter the circulation, and disseminate into the end organs, the disease becomes systemic and patients now harbor many other types of cancer cells such as **circulating tumor cells (CTCs)**, **disseminated tumor cells (DTCs), circulating cancer associated macrophage-like cells (CAMLs)**, and **cancer hybrid cells (CHCs)**. The overarching goal of *Cancer Heterogeneity and Plasticity* is to publish high-rigor research articles, reviews and perspectives spanning the dynamic realm of cancer cell heterogeneity and meticulously dissecting the heterogeneous cancer cell populations.

Another goal of *Cancer Heterogeneity and Plasticity* is to showcase mechanistic research that elucidates the molecular underpinnings of the ‘evil twin’ of cancer cell heterogeneity, *i.e.*, plasticity. Cancer cell plasticity addresses the adaptability of and inter-relationship between/among the heterogeneous cancer cell populations exemplified above. Cancer cell plasticity encompasses cellular and lineage plasticity, the former referring to cellular ‘transformation’ along the same cell lineage whereas the latter to the crossing of cell lineage boundaries. Cancer cell plasticity may be engendered by genetic mutations (e.g., *Rb1*, *p53*), epigenetic alterations, tumor microenvironment (TME) changes (e.g., hypoxia, inflammatory cytokines, *etc.*), and therapeutic interventions [11]. One of the best examples to illustrate both cellular and lineage plasticity is the AR^+^ prostate cancer (PCa) treated with antiandrogens, where castration-resistant PCa (CRPC) may manifest as AR^−^ adenocarcinoma (*i.e.*, CRPC-adeno), which maintains the epithelial lineage, or as AR^−^ neuroendocrine (NE) type of CRPC (*i.e.*, CRPC-NE), which has switched lineage from epithelial to NE. Treatment-induced cancer cell plasticity or reprogramming represents a major nongenetic mechanism whereby cancer cells escape targeted and IO drugs. Cancer cell plasticity exponentially increases the cancer cell heterogeneity and complexities and dramatically reshapes the cancer ecosystem. Emerging evidence suggests that the kaleidoscope of seemingly divergent cancer cell types may represent different developmental states of the same or overlapping cancer cell continuum.

Regardless, both cancer cell heterogeneity and plasticity play important roles in and make significant contributions to development of therapy resistance and cancer metastasis. In-depth understanding of the two inter-twined cancer biology processes should lead to novel therapeutic targets and strategies to tackle them. The scope of *Cancer Heterogeneity and Plasticity* encompasses a wide spectrum of topics, including but not limited to, the elucidation of evolutionary relationship of cancer cell subpopulations (*i.e.*, heterogeneity), molecular mechanisms driving cancer cell plasticity, epigenetic underpinnings of cancer cell reprogramming (e.g., treatment-induced NE transdifferentiation), and reciprocal interactions between and co-evolution of the TME and cancer cell subpopulations. It also extends to embrace studies exploring the impact of the microenvironment and systemic host factors (e.g., hormones, growth factors, inflammatory cytokines, and nerves and neurotrophic factors) on cancer progression, therapeutic resistance, cancer cell dissemination and dormancy, metastasis, and the development of innovative targeted and combinatorial therapies.

*Cancer Heterogeneity and Plasticity* welcomes contributions from various disciplines including cancer, cell, molecular, computational and systems biology, genetics, clinical oncology, bioinformatics, immunology, tumor imaging, and artificial intelligence (AI) and machine learning. The journal is particularly interested in studies that employ and integrate state-of-the-art experimental models (e.g., lineage tracing, organoids), cutting-edge techniques (such as cellular bar-coding, high-fidelity cell lineage/state reporter systems, single-cell multi-omics, quantitative high-content and multiplexed imaging analysis, and spatial transcriptomics/proteomics/pathology), high-throughput CRISPR and drug combination [12,13] screening, and utilization of human cancer specimens (archived or live human tumor samples, PDX, CTCs, ctDNA, *etc.*) to elucidate cancer cell heterogeneity and plasticity. Additionally, contributions shedding light on translating these findings into clinical applications, such as predictive, prognostic and treatment response biomarkers, personalized medicine, and novel intervention strategies, are also highly encouraged.
